# Supplementary material for: Ablation of Sphingosine Kinase 1 Protects Cornea from Neovascularization in a Mouse Corneal Injury Model
Source: Cells. 2022 Sep 17;11(18):2914. doi: 10.3390/cells11182914 (PMC9497123; doi:10.3390/cells11182914)
Supplement: Supplementary file 1 [file cells-11-02914-s001.zip › cells-1827578-supplementary.pdf]

cells

MDPI

Supplementary Materials

Ablation of Sphingosine kinase 1 protects cornea from neovascularization in a mouse corneal injury model

Joseph L. Wilkerson [1,2,†], Sandip K. Basu [3,†], Megan A. Stiles [1], Amanda Prislovsky [3], Richard C. Grambergs [3], Sarah E. Nicholas [4,5], Dimitrios Karamichos [4,5,6], Jeremy C. Allegood [7], Richard L. Proia [8] and Nawajes Mandal [1,3,9,10,*]

[1] Dean A. McGee Eye Institute, University of Oklahoma Health Sciences Center, Oklahoma City, OK 73104, USA; joseph.wilkerson@hsc.utah.edu (J.L.W.); Megan-Stiles@ouhsc.edu (M.A.S.)
[2] Department of Nutrition and Integrative Physiology, University of Utah, Salt Lake City, UT 84112, USA;
[3] Department of Ophthalmology, University of Tennessee Health Sciences Center, Hamilton Eye Institute, Memphis, TN 38163, USA; sbasu8@uthsc.edu (S.K.B.); aprislo1@uthsc.edu (A.P.); rgramber@uthsc.edu (R.C.G.);
[4] North Texas Eye Research Institute, University of North Texas Health Science Center, Fort Worth, TX 76107, USA; sarah.nicholas@unthsc.edu (S.E.N.); dimitrios.karamichos@unthsc.edu (D.K.)
[5] Department of Pharmaceutical Sciences, University of North Texas Health Science Center, Fort Worth, TX 76107, USA;
[6] Department of Pharmacology and Neuroscience, University of North Texas Health Science Center, Fort Worth, TX 76107, USA;
[7] Department of Biochemistry and Molecular Biology, Virginia Commonwealth University School of Medicine, Richmond, VA 23298, USA; jeremy.allegood@vcuhealth.org
[8] Genetics of Development and Disease Branch, National Institute of Diabetes and Digestive and Kidney Diseases, National Institutes of Health, Bethesda, MD 20892, USA; richard.proia@nih.gov
[9] Departments of Anatomy and Neurobiology, and Pharmaceutical Sciences, University of Tennessee Health Sciences Center, Memphis, TN 38163, USA
[10] Memphis VA Medical Center, Memphis, TN 38104, USA
* Correspondence: nmandal@uthsc.edu
† These authors contributed equally to this work.

Supplementary Figure S1

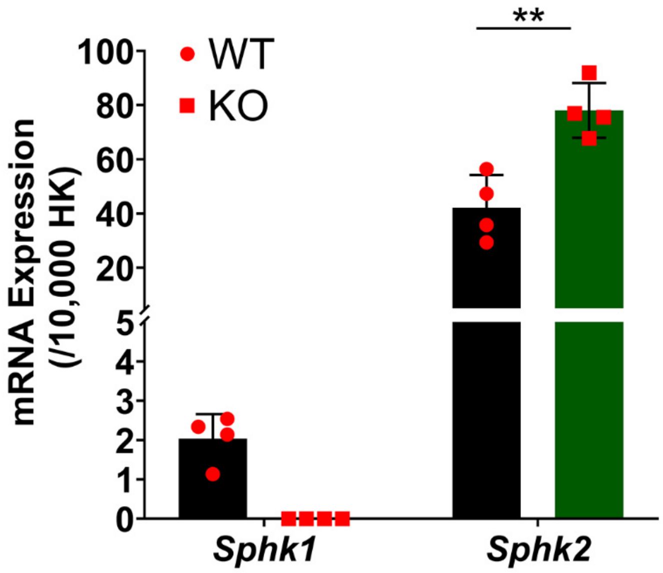

Figure S1. Gene expression of *Sphk1* and *Sphk2* in WT *and Sphk1*$^{-/-}$ mice cornea. Expression level of *Sphk1* mRNA is undetectable in *Sphk1*$^{-/-}$ mice cornea. The expression level of *Sphk2* mRNA is significantly increased in *Sphk1*$^{-/-}$ mice cornea as compared to the WT (Values are Mean ± SD; t-test: **$p \leq 0.01$).

Supplementary Figure S2

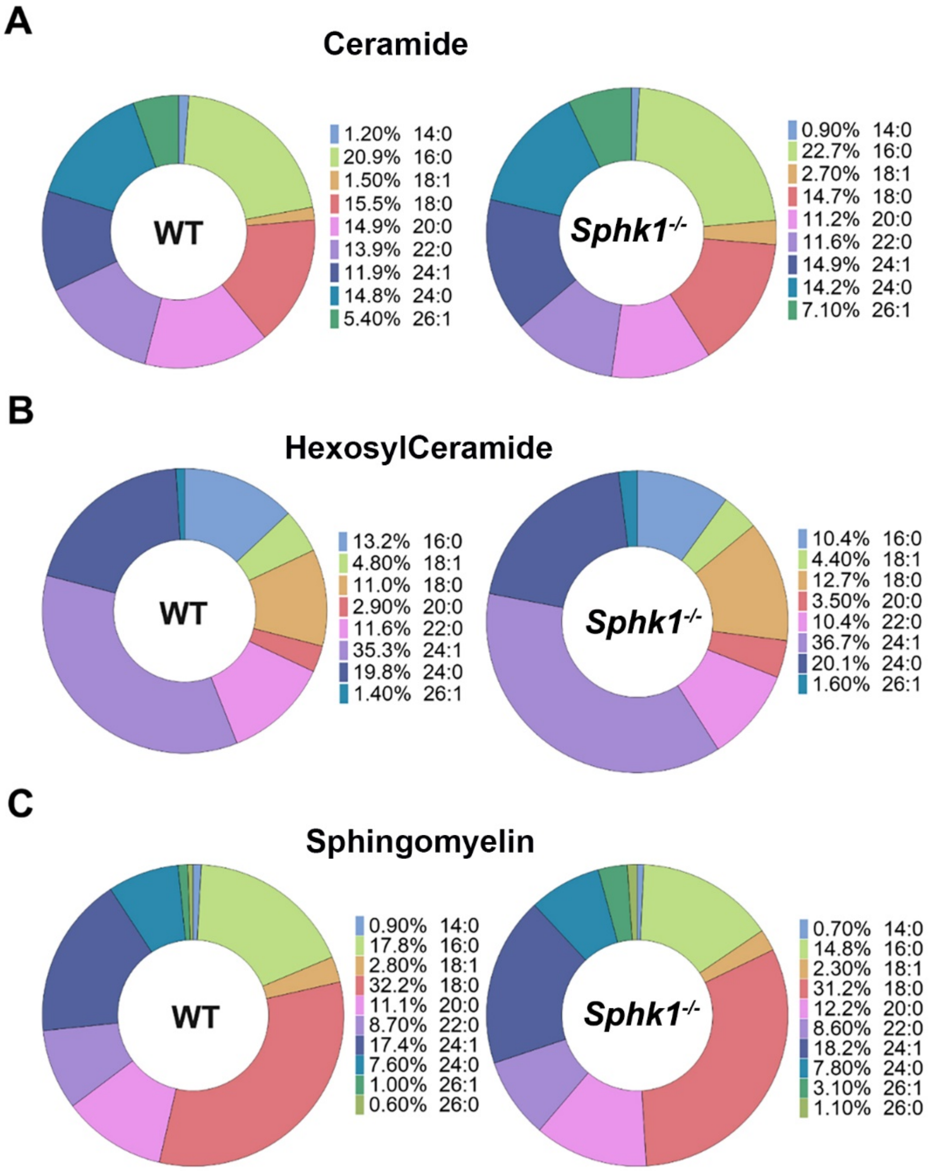

Figure S2. Mole percent composition of sphingolipids in the cornea. Analysis of sphingolipids from WT and *Sphk1*$^{-/-}$ mice cornea (n=6) showing mole percent composition of (A) ceramide, (B) HexCer and (C) sphingomyelin. (Values are mean ± SD).

Supplementary Figure S3

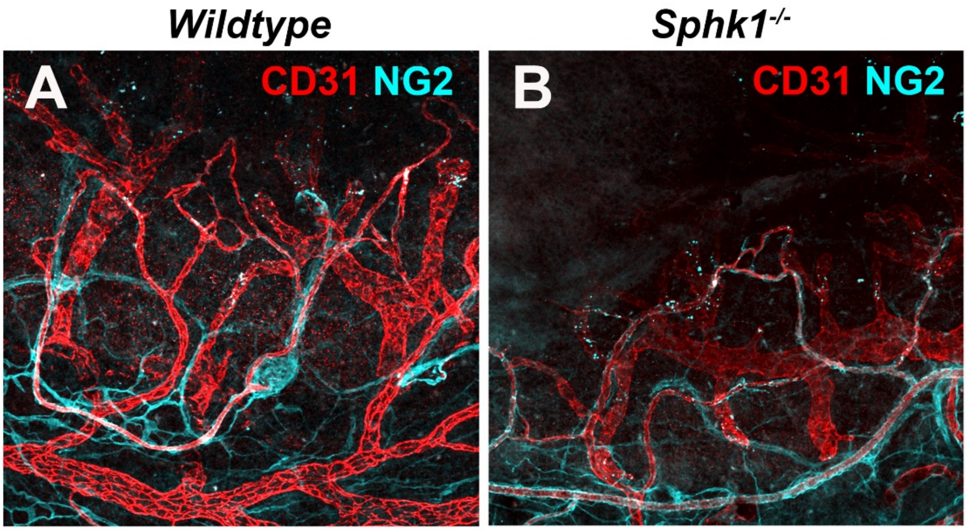

Figure S3. Ablation of *Sphk1* does not affect pericyte migration. Following alkali burn WT (A) and *Sphk1*$^{-/-}$ (B) mice corneas were collected on 7 PBD and flat mounted (n=10). Immunostaining with CD31 (red) for blood vessels and NG2 (cyan), a marker for pericytes, shows that pericytes are being recruited normally to the newly forming vessels with no difference in recruitment between WT and *Sphk1*$^{-/-}$ mice.

Supplementary Figure S4

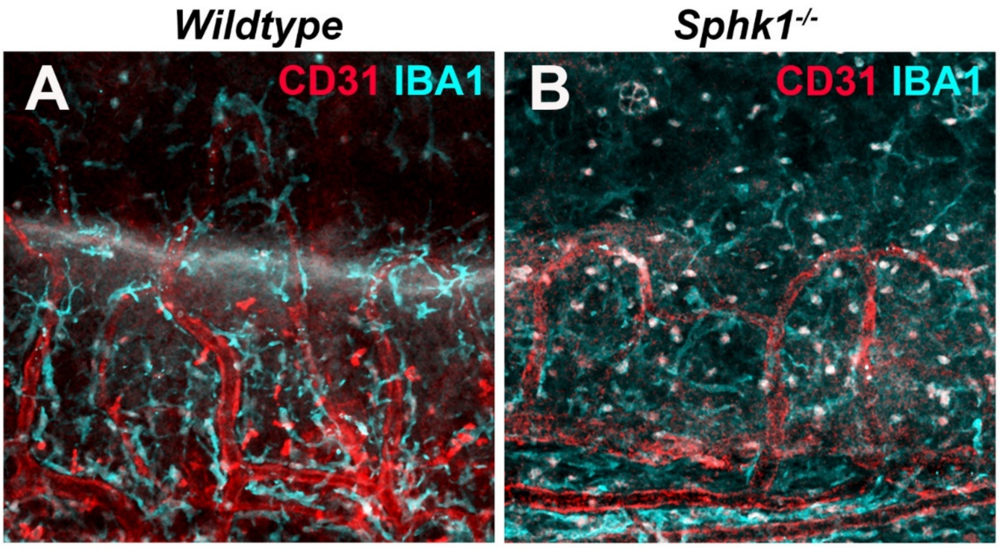

Figure S4. **Ablation of *Sphk1* does not affect macrophage egress from blood vessels.** WT (A) and *Sphk1*$^{-/-}$ (B) mice corneas were subjected to alkali burn (n=8). Corneas were harvested on 7 PBD, flat mounted and immuno-stained with CD31 (red) for blood vessels and IBA1 (cyan) for macrophage. Macrophage egress can be observed from vessels and no significant difference was noticed between WT and *Sphk1*$^{-/-}$ corneas.

Supplementary Figure S5

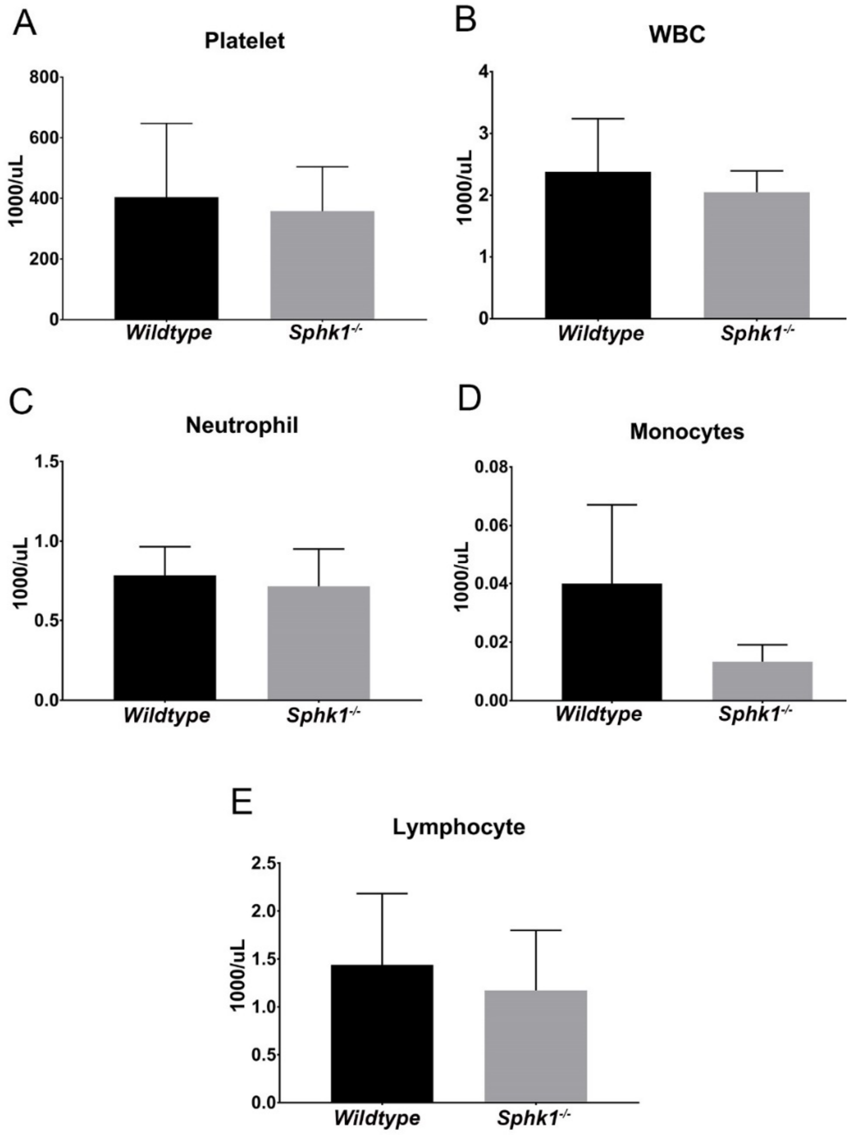

Figure S5. Complete blood counts showed that reduced plasma S1P has no effect on major classes of immune cells. (A) Platelets remain unaffected. (B) White blood cell levels remain unaffected even with reduced S1P. (C) Neutrophils remain unaffected. (D) Monocyte numbers show a reduction trend but is not significant. (E) Lymphocytes remain unaffected. (n=4, Values are Mean ± SD; For A, B, C, and E: two-tailed t-test, $p \leq 0.05$; For D: Mann-Whitney two-tailed test, U=0.5).
